# Clinical Characteristics and Treatment Response With Checkpoint Inhibitors in Malignant Melanoma of the Vulva and Vagina

**DOI:** 10.1097/LGT.0000000000000583

**Published:** 2020-11-30

**Authors:** Christoph Wohlmuth, Iris Wohlmuth-Wieser, Stéphane Laframboise

**Affiliations:** 1 Division of Gynecologic Oncology, Department of Surgical Oncology, University Health Network, Toronto, Ontario, Canada; 2 Department of Obstetrics and Gynecology, University of Toronto, Ontario, Canada; 3 Department of Obstetrics and Gynecology, Paracelsus Medical University, Salzburg, Austria; 4 Division of Dermatology, Department of Medicine, University of Toronto, Ontario, Canada; 5 Department of Dermatology and Allergology, Paracelsus Medical University, Salzburg, Austria

**Keywords:** melanoma, vulva, vagina, vulvovaginal melanoma, checkpoint inhibitors, immunotherapy

## Abstract

The aims of the study were to assess the clinical and histopathological characteristics of a comprehensive cohort of women with vulvovaginal melanoma (VVM) treated at our institution and to study the treatment response of checkpoint inhibitors in this patient cohort.

**Materials and Methods:**

This is a retrospective study of women with invasive VVM treated at the Princess Margaret Cancer Centre in Toronto, Ontario, Canada, over a period of 15 years. Clinical and histopathological characteristics, treatment, as well as treatment-related outcome were analyzed in 32 women. Treatment response was evaluated retrospectively using the “response criteria for use in trials testing immunotherapeutics” (iRECIST). The objective response rate was defined as the proportion of patients with complete or partial response based on the best overall response.

**Results:**

At a median follow-up of 37.8 months (5.8–110.4), 26 women (81.3%) had disease progression and 16 (50%) died. Thirteen patients with locally unresectable or metastatic melanoma were treated with immune checkpoint inhibitors. Ten additional cases were identified from previously published reports. The best objective response rate for immune checkpoint inhibitors was 30.4% (95% CI = 11.6%–49.2%) and the clinical benefit rate was 52.2% (95% CI = 31.8%–72.6%). The clinical benefit rate was significantly better for programmed cell death protein 1 inhibitors (or a combination) compared with ipilimumab alone (Fisher exact, *p* = .023). Grade 3/4 adverse events were observed in 3 (13.0%) of the 23 patients.

**Conclusions:**

Women with VVM constitute a high-risk group with poor overall prognosis. Immune checkpoint inhibitors are effective in the treatment of metastatic melanoma in this patient cohort.

Melanomas of the vulva and vagina account for 1% of all melanomas diagnosed in women and for 5.3% of all vulvar and 5.5% of all vaginal malignancies.^1^

Genital tract melanomas are commonly categorized as mucosal melanomas, but this has been questioned by studies showing different mutational characteristics suggesting that vulvovaginal melanomas (VVMs) may be classified as a unique subclass.^2–5^

Data on VVMs are scarce, and to date, only 1 prospective study has been completed: The Gynecologic Oncology Group 73 protocol suggested that the American Joint Committee on Cancer (AJCC) staging was the best predictor for survival and Breslow’s depth of invasion and lymphovascular space invasion were predictive of lymph node metastases.^6^ In a population-based study, we have recently shown that VVMs have a particularly poor prognosis with a median overall survival of 53 months in vulvar melanoma and 16 months in vaginal melanoma with no important change in survival over time.^1^

The treatment landscape of advanced and metastatic melanoma has drastically changed with the introduction of immune checkpoint inhibitors. Trials with the cytotoxic T-lymphocyte–associated protein 4 (CTLA-4) inhibitor ipilimumab and the programmed cell death protein 1 (PD-1) inhibitors nivolumab and pembrolizumab have shown profound improvements of survival in patients with unresectable or metastatic melanoma.^7–9^ In a pooled analyses of clinical trials, mucosal melanomas, however, had lower response rates to nivolumab and pembrolizumab compared with cutaneous melanomas.^10,11^ Data for VVMs are scarce.

The aims of this study are to describe clinical characteristics of a comprehensive cohort of women with VVM treated at our institution and to assess the treatment response of immune checkpoint inhibitors in this patient cohort.

## METHODS

### Study Population

This is a retrospective single-center cohort study of women with invasive melanoma of the vulva or vagina treated at the Princess Margaret Cancer Centre in Toronto, Ontario, Canada. The study protocol was approved by the institutional review ethics board (UHN 19-5620). All women with histologically confirmed invasive vulvar or vaginal melanoma diagnosed over a period of 15 years (2004–2018) were included and all cases were reviewed by an expert pathologist at the time of initial presentation; women with melanoma in situ without invasive components were not included. Vulvar melanomas were staged according to the AJCC staging classification in the eighth edition, and vaginal melanomas were classified as local, regional, or distant.^12^ Demographic data, Eastern Cooperative Oncology Group performance (ECOG) score, histopathology, type of surgery, lymph node assessment, adjuvant treatment, recurrence, treatment details for recurrent disease, and vital status were extracted from the electronic patient records. Programmed death-ligand 1 was not routinely tested in our patient cohort. Selection for immunotherapy was based on availability. Treatment response was evaluated retrospectively using the “response criteria for use in trials testing immunotherapeutics” (iRECIST).^13^ The objective response rate (ORR) was defined as the proportion of patients with complete (iCR) or partial response (iPR) based on the best overall response (iBOR).^13^ The clinical benefit rate (CBR) was defined as the proportion of patients with iCR, iPR, or stable disease (iSD). An iSD was assigned if no disease progression occurred for at least 2 months. Adverse events were categorized using the common terminology criteria for adverse events version 5.0.^14^ The treatment response of immune checkpoint inhibitors was analyzed in our cohort. In addition, treatment response from previously published case series and case reports was identified from PubMed using the search terms “ipilimumab,” “nivolumab,” or “pembrolizumab” in combination with “vulva” or “vagina,” and a separate analysis was performed for our cohort and the combined cohort. Reports without information on treatment response were not included.

### Statistical Analyses

Descriptive statistics was used to report demographic data. Continuous variables were compared using the Student *t* test, Mann-Whitney test, or Wilcoxon test, as appropriate. Categorical data were compared using the Fisher exact test. Progression-free survival (PFS) and overall survival (OS) for the comprehensive cohort were calculated from the date of diagnosis to date of progression or death (PFS) and date of diagnosis to date of death (OS). Progression-free survival and OS for the subgroup analysis of immune checkpoint inhibitors were calculated from the date of treatment initiation to the date of progression or death, respectively. The Kaplan-Meier method with log-rank test was used to analyze PFS and OS. The 2- and 5-year survival rates were calculated using the Kaplan-Meier method. Statistical analysis was performed using SPSS Version 26, IBM, Armonk. A *p* value of less than .05 was considered statistically significant, all tests were 2-sided.

### Role of the Funding Source

I.W.-W. is supported by a grant from the Austrian Science Fund (Project Number J 4382-B) to fund her fellowship at the Division of Dermatology, Department of Medicine, University of Toronto, Ontario, Canada. No external funding was used in the preparation of this manuscript.

## RESULTS

### Patient Characteristics

In total, 32 women with invasive vulvar (*n* = 28) and vaginal (*n* = 4) melanoma were treated at our institution over a period of 15 years and included in our study. Demographic and clinical characteristics are shown in Table 1. The mean age at diagnosis was 66 years, and a significant proportion of patients was diagnosed with advanced disease stage, tumor thickness of greater than 4 mm, ulcerations, and high mitotic count; 31.3% already reported symptoms from melanoma including bleeding, pruritus, and pain at the time of diagnosis. Histologic characteristics are shown in Table 2. BRAF was tested in 25 patients and was positive in 2 (8.0%), cKIT was positive in 3 (13.6%) of the 22 patients tested, and NRAS mutations were detected in 2 (13.3%) of the 15 patients tested. A mutation in SF3B1 was found in 2 patients and 1 woman was found to have a PTEN mutation.

**TABLE 1:** Patient Characteristics

Parameter	
Age at diagnosis, y	
Mean ± SD	66.3 ± 14.0
Median (range)	66.0 (40–96)
Pregnancy history	
Gravida	2 (0–3)
Para	2 (0–3)
ECOG performance status at diagnosis	
ECOG 0	23 (71.9%)
ECOG 1	6 (18.8%)
ECOG 2	2 (6.3%)
ECOG 3	1 (3.1%)
History of previous malignancy	
History of melanoma	2 (6.3%)
History of other malignancy	7 (21.9%)
Tumor stage at diagnosis	
Vulvar melanoma (*n* = 28)	
AJCC stage I	1 (3.6%)
AJCC stage II	13 (46.4%)
AJCC stage III	11 (39.3%)
AJCC stage IV	3 (10.7%)
Vaginal melanoma (*n* = 4)	
local	0 (0%)
regional	4 (100%)
distant	0 (0%)
Reported symptoms	
Any symptoms reported	10 (31.3%)
Pruritus	4 (12.5%)
Bleeding	8 (25.0%)
Pain	4 (12.5%)
Organ involvement	
Labia majora	22 (68.8%)
Labia minora	15 (46.9%)
Clitoris	10 (31.1%)
Urethra	4 (12.5%)
Anus	0 (0%)
Surgery	
Radical local excision	31 (96.9%)
Exenteration	1 (3.1%)
Surgical lymph node assessment	
Performed	27 (84.4%)
Nodal metastases	14 (51.9%)
Negative lymph nodes	13 (48.1%)

**TABLE 2:** Histologic Characteristics

Characteristics	
Tumor thickness, mm	
Median (range)	8 (1.1–68)
≤1.00	0 (0%)
1.01–2.00	4 (12.9%)
2.01–4.00	7 (22.6%)
>4.00	20 (62.5%)
Ulceration	
Present	24 (77.4%)
Absent	7 (21.9%)
Mitotic count, mitoses/mm^2^	
Median (range)	8 (0–50)
0	1 (3.4%)
1	1 (3.4%)
2–10	14 (48.3%)
>10	13 (44.8%)

All women underwent surgery, and the lymph node status was surgically evaluated in 84.4% of all patients and in 88.0% of those with nonmetastatic vulvar melanoma. Adjuvant systemic treatment was given in 5 patients (15.6%): adjuvant interferon α in 3 and nivolumab in 2 patients, and their outcome is reported hereinafter.

### Outcome

At a median follow-up of 37.8 months (5.8–110.4), 26 (81.3%) women had disease progression and 16 (50%) died. The median PFS was 17.7 months (95% CI = 5.5–29.8 months), and the 2- and 5-year PFS rates were 35.4% and 23.2%, respectively. The median OS was 59.1 months (95% CI = 23.6–94.5 months), and the 2- and 5-year OS rates were 71.1% and 45.6%, respectively. The 2-year PFS rate by the AJCC stage in vulvar melanoma was as follows: stage I, 100%; stage II, 35.9%; stage III, 42.4%; stage IV, 33.3%; and in the 4 patients with regional vaginal melanoma, 0% (*p* = .126).

Fifteen (51.7%) of the 29 nonmetastatic patients at diagnosis developed distant metastases with a median time to metastatic disease of 39.5 months (95% CI = 0–84.2 months). Two patients received adjuvant nivolumab: 1 patient with vaginal melanoma developed brain metastases during adjuvant treatment with nivolumab. She was treated with stereotactic radiation and switched to pembrolizumab. The best overall response was iSD, but she ultimately progressed and died of melanoma (see Table 3, PMH05). The second patient receiving adjuvant nivolumab had vulvar melanoma AJCC stage IIIC. She had a local recurrence after 77 months, which was excised, and she has now been recurrence-free for 8 months.

**TABLE 3:** Characteristics of Women With Malignant Melanoma of the Vulva or Vagina Receiving Immune Checkpoint Inhibitors (Combining Our Own Patient Cohort and Patients Identified From the Literature)

Patient	Site	Stage at treatment initiation*^a^* (metastases)	Prior systemic therapy	Prior XRT, Site	Immunotherapy	iBOR	PFS*^b^*	irAEs	OS*^c^*	Vital status
PMH01	Vulva	IIIC, unresectable	None	None	Pembrolizumab	iCPD	2	None	18	Alive with disease
PMH02	Vulva	IV (lung)	None	None	Ipilimumab + nivolumab	iSD	18	Uveitis G1, peripheral sensory neuropathy G3	18	Alive with disease
PMH03	Vulva	IV (liver)	None	None	Ipilimumab + nivolumab	iCPD	1	None	1	Died of disease
PMH04	Vulva	IV (liver)	None	None	Nivolumab	iPR	15	Hepatitis G1	15	Alive with disease
PMH05	Vagina	Distant (brain)	Nivolumab, adjuvant	None	Pembrolizumab	iSD	4	None	16	Died of disease
PMH06	Vulva	IV (lung)	None	None	Ipilimumab	iCR	56	None	56	Alive with NED
PMH07	Vulva	IV (lung, liver)	Interferon, adjuvant	None	1. Ipilimumab	iCPD	3	Maculopapular exanthema G1, Hepatitis G1	17	Died of disease
					2. Pembrolizumab	iSD	4	None		
PMH08	Vulva	IV (liver)	None	Liver	1. Ipilimumab	iCPD	3	Maculopapular exanthema G1	50	Alive with disease
					2. Pembrolizumab	iPR	9	None		
PMH09	Vulva	IV (lung, brain)	Carboplatin/paclitaxel	Brain	Ipilimumab	iCPD	3	None	6	Died of disease
PMH10	Vulva	IV (lung)	Dacarbazine	Groin	1. Ipilimumab	iCPD	3	None	87	Alive with NED
					2. Pembrolizumab	iCR	77	Hyperthyroidism G2, DM G3, Erythema nodosum G1		
PMH11	Vulva	IV (lung, bone)	None	Vulva + groin	Ipilimumab	iCPD	1	None	1	Died of disease
PMH12	Vulva	IV (lung, abdomen)	Carboplatin/paclitaxel	Vulva + groin	1. Ipilimumab	iCPD	3	None	16	Died of disease
					2. Pembrolizumab	iCPD	3	None		
PMH13	Vulva	IV (lung, abdomen, soft tissue)	Carboplatin/paclitaxel	Abdomen + groin	Ipilimumab	iSD	2	None	13	Died of disease
Indini1^15^	Vulva	IV (lung)	CVD	None	Ipilimumab	iCPD	4	None	7	Died of disease
Indini2^15^	Vulva	IV (lung, bone)	None	None	Pembrolizumab	iPR	10	Arthralgia G2, hypothyroidism G2	10	Alive with disease
Indini3^15^	Vagina	Distant (liver)	None	None	Pembrolizumab	iCPD	2	None	4	Alive with disease
Indini4^15^	Vagina	Distant (n.s.)	None	Vagina	Nivolumab	iSD	4	Cutaneous rash G1	4	Alive with disease
Indini5^15^	Vagina	Distant (liver, pancreas, soft tissues, bone)	None	None	Ipilimumab	iCPD	3	None	7	Died of disease
Indini6^15^	Vagina	Distant (lung)	Dacarbazine	None	Ipilimumab	iCPD	3	None	18	Died of disease
Daix1^16^	Vagina	Regional, unresectable	None	None	Nivolumab	iCR	8	Pruritus G1	8	Alive with NED
Anko1^17^	Vagina	Distant (liver, lung, bone)	None	None	Nivolumab	iPR/iCR	17	Thyroiditis, n.s.	17	Alive
Komatsu-Fujii1^18^	Vagina	Distant (lung)	None	None	1. Nivolumab	iCPD	n.s.	n.s.	n.s.	Alive with disease
					2. Pembrolizumab	iCPD	n.s.	n.s.		
					3. Ipilimumab	iCPD	n.s.	n.s.		
Inoue1^19^	Vagina	Distant (brain)	None	Brain	Nivolumab	iCPD	2	Hepatitis G3	n.s.	Alive with disease

Characteristics of the 13 patients treated our institution (PMH1–PMH13) and 10 additional previously published cases.

*^a^*Stage at initiation of treatment with immune checkpoint inhibitor, AJCC stage for vulvar melanomas and local/regional/distant for vaginal melanomas.

*^b^*PFS in months defined from treatment initiation with immune checkpoint inhibitor to date of progression or death.

*^c^*OS in months defined from treatment initiation with first immune checkpoint inhibitor to date of last follow-up or death.

CVD indicates cisplatin-vinblastine-dacarbazine; DM, diabetes mellitus; G, grade; iCPD, confirmed progressive disease; irAEs, immune-related adverse events; NED, no evidence of disease; n.s., not specified; XRT, radiation therapy.

### Treatment Response of Immune Checkpoint Inhibitors in Unresectable or Metastatic Melanoma

Thirteen patients with locally unresectable or metastatic melanoma were treated with immune checkpoint inhibitors, and 4 patients were initially treated with ipilimumab and switched to a PD-1 inhibitor after treatment failure. The best overall ORR with immunotherapy in the 13 patients was 30.8% (95% CI = 5.7%–55.9%), and the CBR was 61.5% (95% CI = 35.1%–88.0%). The median PFS was 4.0 months (95% CI = 2.3–5.7 months), and the median OS was 17.0 months (95% CI = 12.7–21.3 months). Ipilimumab was given in 8 patients; the ORR was 12.5% (95% CI = 0%–35.4%), and the CBR was 25.0% (95% CI = 0%–55.0%). Programmed cell death protein 1 inhibitors or a combination of CTLA-4 and PD-1 inhibitors were given in 9 patients; the ORR was 33.3% (95% CI = 2.5%–64.1%), and the CBR was 66.7% (95% CI = 35.9%–97.5%).

In addition, 13 patients with VVM receiving immune checkpoint inhibitors were identified from previously published cases in the literature^15–20^; 10 patients with metastatic or unresectable VVM were included (see Table 3); 3 patients, who received neoadjuvant ipilimumab and radiation and subsequently underwent surgery, were not included.^20^ The best overall ORR with immune checkpoint inhibitors in the combined cohort of the 23 patients was 30.4% (95% CI = 11.6%–49.2%), and the CBR was 52.2% (95% CI = 31.8%–72.6%). The median PFS was 4.0 months (95% CI = 2.7–5.3 months), and the median OS was 17.0 months (95% CI = 12.7–21.3 months). The ORR for ipilimumab alone was 8.3% (95% CI = 0%–24%) compared with 37.5% (95% CI = 13.8%–61.2%, Fisher exact, *p* = .184) for PD-1 inhibitors or a combination of CTLA-4 and PD-1 inhibitors. The CBR was 16.7% (95% CI = 0%–37.8%) for ipilimumab compared with 62.5% (95% CI = 38.8%–86.2%, Fisher exact, *p* = .023) for PD-1 inhibitors or a combination of CTLA-4 and PD-1 inhibitors. The median PFS for ipilimumab alone was 3.0 months (95% CI = 2.6–3.4 months) compared with 9.0 months (95% CI = 1.9–16.1 months, *p* = .062) for PD-1 inhibitors or the combination of CTLA-4 and PD-1 inhibitors. Severe adverse events (grade 3/4) were observed in 2 (15.4%) of the 13 patients in our cohort and 3 (13.0%) of the 23 patients in the total cohort.

## DISCUSSION

In this study, we report the clinical characteristics of VVM and the treatment response to immune checkpoint inhibitors in a comprehensive cohort.

Most women were diagnosed in advanced disease stages with poor prognostic indicators. Half of the nonmetastatic patients undergoing surgical lymph node assessment had lymph node metastases, and most our patients had a high mitotic count, both of which were recently shown to be important independent predictors for survival in women with VVMs.^1,21^ More than 80% of our cohort had disease recurrence or progression with a 2- and 5-year PFS rate of 35.4% and 23.2%, respectively. More than 50% of the women, who were free of distant metastases at diagnosis, developed metastatic disease. Therefore, women with VVM represent a high-risk group.

Consistent with previous reports and unlike in cutaneous melanomas, only a small proportion of patients had BRAF mutations, limiting the treatment options with BRAF/MEK inhibitors.^3,4^ cKIT mutations were observed 14% and NRAS mutations in 13%; 2 patients were found to have a mutation in SF3B1, a mutation that was recently found to be more prevalent in VVMs and may be associated with worse outcome.^22^

**FIGURE 1:**
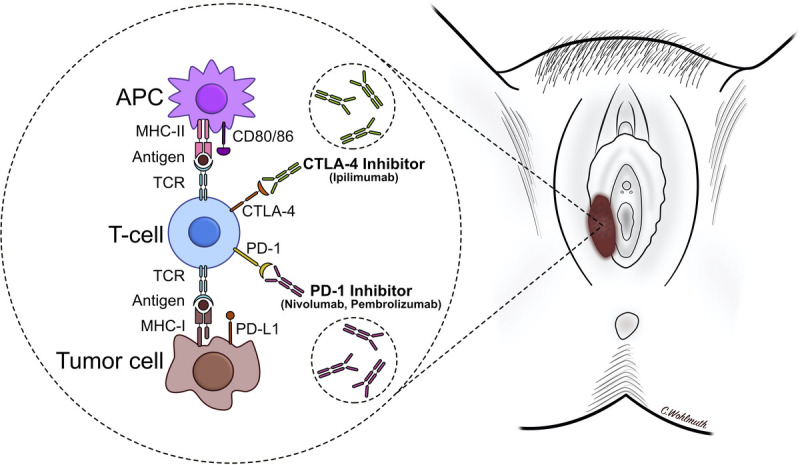
Mechanism of action of immune checkpoint inhibitors in malignant melanoma of the vulva and vagina. Antigen presenting cells present the tumor antigen to T cells through MHC-II but also express inhibitory signals (CD80/86). CD80/86 binds to CTLA-4 and prevents T-cell activation. Ipilimumab, a monoclonal antibody that binds and inhibits CTLA-4, prevents this inhibition resulting in T-cell activation. Similarly, tumor cells express PD-L1 that binds to PD-1 receptors expressed on T cells, resulting in T-cell anergy. nivolumab and pembrolizumab (PD-1 inhibitors) bind PD-1 resulting in T-cell activation. APC, antigen presenting cells; CTLA-4, cytotoxic T-lymphocyte–associated antigen 4; MHC-I, major histocompatibility complex 1; MHC-II, major histocompatibility complex 2; PD-L1, programmed death-ligand 1; TCR, T-cell receptor.

The introduction of immune checkpoint inhibitors has led to an enormous progress in melanoma treatment and checkpoint inhibitors are now United States Food and Drug Administration and European Medicines Agency approved in the adjuvant and metastatic setting. The mechanism of action of CTLA-4 and PD-1 inhibitors is shown in Figure 1. For ipilimumab, we have observed an ORR of 8.3% and a CBR of 16.7% with median PFS of 3.0 months. The ORR is notably lower compared with 21.2% in cutaneous melanoma but identical to the recently reported response rate in mucosal melanomas combining data from 6 clinical trials (2 phase I trials: CA209-003^23^ and CA209-038^24^; 1 phase II trial: CheckMate 069^25^; and 3 phase III trials: CheckMate 066,^26^ CheckMate 037,^27^ and CheckMate 067^8,10^).

For PD-1 or a combination of PD-1 and CTLA-4 inhibitors, the ORR was 37.5% and the CBR was 62.5% with a median PFS of 9.0 months. This is again lower compared with the ORR of 60.4% reported for a combination of nivolumab and ipilimumab^10^ but comparable with 40.9% for nivolumab^10^ and 33.0% for pembrolizumab (combining 3 clinical trials, KEYNOTE-001,^28^ KEYNOTE-002,^29^ and KEYNOTE-006^9,11^ in cutaneous melanoma). Severe adverse events were observed in 15.4% of our cohort, which is comparable with the rate observed in mucosal melanomas.^10^

### Strengths and Limitations

This study investigates a large series of well-described cases of vulvar and vaginal melanoma diagnosed and treated at a comprehensive cancer center. We report clinical characteristics, outcome and treatment response with immune checkpoint inhibitors. The study is, however, limited by its retrospective design. Furthermore, including case reports into the analysis of treatment response adds the risk of publication bias. We have therefore analyzed our own series including all patients with VVM treated at our institution separately, and no distortion of the response rates was observed when adding the additional 10 cases previously published in the literature.

## CONCLUSIONS

Women with VVM constitute a high-risk group with poor overall prognosis. Immune checkpoint inhibitors are effective with a complete or partial response being observed in approximately one third of women with locally unresectable or metastatic VVM. Programmed cell death protein 1 inhibitors or a combination of CTLA-4 and PD-1 inhibitors were associated with a significantly higher CBR and a trend toward longer progression-free survival compared with CTLA-4 inhibitors alone.
